# Diagnostic value of lasik test for detection of lumbar discopathy in patients attending emergency department with acute low back pain

**Published:** 2012-11

**Authors:** Alireza Aala, Saeid Alizadeh Shahri, Ali Meshkini, Samad Shams Vahdati

**Affiliations:** ^*a*^Emergency Department , Imam Reza Hospital, Faculty of Medicine, Tabriz University of Medical Sciences, Tabriz, Iran.; ^*b*^Neurosurgery Department, Imam Reza Hospital, Faculty of Medicine, Tabriz University of Medical Sciences, Tabriz, Iran.

## Abstract

**Background:**

Low back pain is one of the most important health problems. Disk herniation is one of the common causes of low back pain. Accurate examination is the best and simplest diagnostic methods. Physical examination, Straight leg raise (SLR) and neurologic tests as well as other tests are usually used to locate the exact place of herniation. Paraclinical studies such as magnetic resonance imaging (MRI) should be used because of the low diagnostic efficiency of physical examination.

**Methods:**

100 patients attended the emergency unit because of back pain or radicular pain, were enrolled in the study. SLR, Reverse- SLR (R-SLR) and Crossed-SLR (C-SLR) tests were performed for all patients. Then, the patients were referred to the neurosurgery unit for treatment. Lumbar MRI was taken for all patients. Then, all patients were followed up and these data were collected.

**Results:**

Sensitivity and specificity of SLR test, C-SLR, and R-SLR tests was 77.52% and 36.36%, 29.21% and 90.9%, and 88.89% and 10.94%, respectively. There was no significant relationship between MRI and positive discopathy in the physical examination and SLR (P=0.11).

**Conclusions:**

SLR test has high sensitivity and low specificity. If the SLR test was accompanied by with physical examination the resulting sensitivity and specificity will increase. Findings of this study showed that SLR test has more sensitivity, while C-SLR has more specificity in low back pain diagnosis.

**Keywords:**

SLR test, Low Back Pain, Discopathy, Straight leg raise


					Volume 4, Suppl. 1 Nov 2012
Publisher: Kermanshah University of Medical Sciences
URL: http://www.jivresearch.org
Copyright:
Congress of Iranian Neurosurgeons, 14-16 November 2012, Kermanshah, Iran
Paper No. 25
Diagnostic value of lasik test for detection of lumbar
discopathy in patients attending emergency department with
acute low back pain
Alireza Aala a
, Saeid Alizadeh Shahri a
, Ali Meshkini b,*
, Samad Shams Vahdati a
a
Emergency Department , Imam Reza Hospital, Faculty of Medicine, Tabriz University of Medical Sciences, Tabriz, Iran.
b
Neurosurgery Department, Imam Reza Hospital, Faculty of Medicine, Tabriz University of Medical Sciences, Tabriz, Iran.
Abstract:
Background: Low back pain is one of the most important health problems. Disk herniation is one of the common
causes of low back pain. Accurate examination is the best and simplest diagnostic methods. Physical examination,
Straight leg raise (SLR) and neurologic tests as well as other tests are usually used to locate the exact place of
herniation. Paraclinical studies such as magnetic resonance imaging (MRI) should be used because of the low
diagnostic efficiency of physical examination.
Methods: 100 patients attended the emergency unit because of back pain or radicular pain, were enrolled in the
study. SLR, Reverse- SLR (R-SLR) and Crossed-SLR (C-SLR) tests were performed for all patients. Then, the patients
were referred to the neurosurgery unit for treatment. Lumbar MRI was taken for all patients. Then, all patients were
followed up and these data were collected.
Results: Sensitivity and specificity of SLR test, C-SLR, and R-SLR tests was 77.52% and 36.36%, 29.21% and
90.9%, and 88.89% and 10.94%, respectively. There was no significant relationship between MRI and positive
discopathy in the physical examination and SLR (P=0.11).
Conclusions: SLR test has high sensitivity and low specificity. If the SLR test was accompanied by with physical
examination the resulting sensitivity and specificity will increase. Findings of this study showed that SLR test has more
sensitivity, while C-SLR has more specificity in low back pain diagnosis.
Keywords:
SLR test, Low Back Pain, Discopathy, Straight leg raise
* Corresponding Author at:
Ali Meshkini: Department Medical Faculty, Tabriz University of Medical Sciences, Tabriz, Iran, Tel: 09141144484,
Email: meshkinia@yahoo.com, (Meshkini A.).

				

